# Role of α7-nicotinic acetylcholine receptor in nicotine-induced invasion and epithelial-to-mesenchymal transition in human non-small cell lung cancer cells

**DOI:** 10.18632/oncotarget.10498

**Published:** 2016-07-08

**Authors:** Chun Zhang, Xu-Ping Ding, Qing-Nan Zhao, Xin-Jie Yang, Shi-Min An, Hao Wang, Lu Xu, Liang Zhu, Hong-Zhuan Chen

**Affiliations:** ^1^ Department of Pharmacology and Chemical Biology, Shanghai Jiao Tong University School of Medicine, Shanghai 200025, China; ^2^ Department of Pharmacy, Xinhua Hospital affiliated to Shanghai Jiao Tong University School of Medicine, Shanghai 200092, China

**Keywords:** α7-nAChR, nicotine, invasion, EMT, NSCLC

## Abstract

Nicotine via nicotinic acetylcholine receptors (nAChRs) stimulates non-small cell lung cancer (NSCLC) cell invasion and epithelial to mesenchymal transition (EMT) which underpin the cancer metastasis. However, the receptor subtype-dependent effects of nAChRs on NSCLC cell invasion and EMT, and the signaling pathway underlying the effects remain not fully defined. We identified that nicotine induced NSCLC cell invasion, migration, and EMT; the effects were suppressed by pharmacological intervention using α7-nAChR selective antagonists or by genetic intervention using α7-nAChR knockdown via RNA inference. Meanwhile, nicotine induced activation of MEK/ERK signaling in NSCLC cells; α7-nAChR antagonism or MEK/ERK signaling pathway inhibition suppressed NSCLC cell invasion and EMT marker expression. These results indicate that nicotine induces NSCLC cell invasion, migration, and EMT; the effects are mediated by α7-nAChRs and involve MEK/ERK signaling pathway. Delineating the effect of nicotine on the NSCLC cell invasion and EMT at receptor subtype level would improve the understanding of cancer biology and offer potentials for the exploitation of selective ligands for the control of the cancer metastasis.

## INTRODUCTION

Lung cancer represents the leading cause of cancer mortality worldwide with non-small cell lung cancer (NSCLC) accounting for over 85% of all lung cancers [1]. The vast majority of patients are diagnosed at the late stage after the onset of cancer metastasis and they die from the distant metastasis rather than the primary cancer [2]. This warrants a need for elucidating the biological mechanism underlying the metastasis and seeking novel therapeutic targets and strategies aiming to inhibit metastasis [3].

Cigarette smoking is the leading risk factor driving lung cancer [4]. Nicotine, as the major addictive component in cigarettes, is reported to not only promote cancer cell survival, proliferation, and angiogenesis, but also contribute to tumor dissemination, invasion, and epithelial to mesenchymal transition (EMT), an essential embryonic process fueling metastatic spread [5–7].

Major effects of nicotine are elicited via its binding to and activation of nicotinic acetylcholine receptors (nAChRs). nAChRs are ligand-gated ion channel proteins comprising various combinations of α1–α10, β1–β4, γ, δ, and ε subunits. Differences in subunit combination determine the distinct functional and pharmacological properties of the receptors that are formed. The activation of different nAChR subtypes results in differential effects. While some lead to growth-promoting cues [6], others have the opposite effects in various tumors [8]. The receptor subtype-dependent effects of nAChRs on NSCLC cell invasion and EMT, and the signaling pathway underlying the effects remain not fully defined. Here, we have identified that nicotine induces NSCLC cell invasion, migration, and EMT; the effects are mediated by α7 homomeric nAChRs (α7-nAChRs) and involve MEK/ERK signaling pathway. Delineating the effect of nicotine on NSCLC cell invasion and EMT at receptor subtype level would improve the understanding of cancer biology and offer potentials for the exploitation of selective ligands for the control of the cancer metastasis.

## RESULTS

### α7-nAChR mediates nicotine-induced NSCLC cell invasion and migration

**Figure 1:**
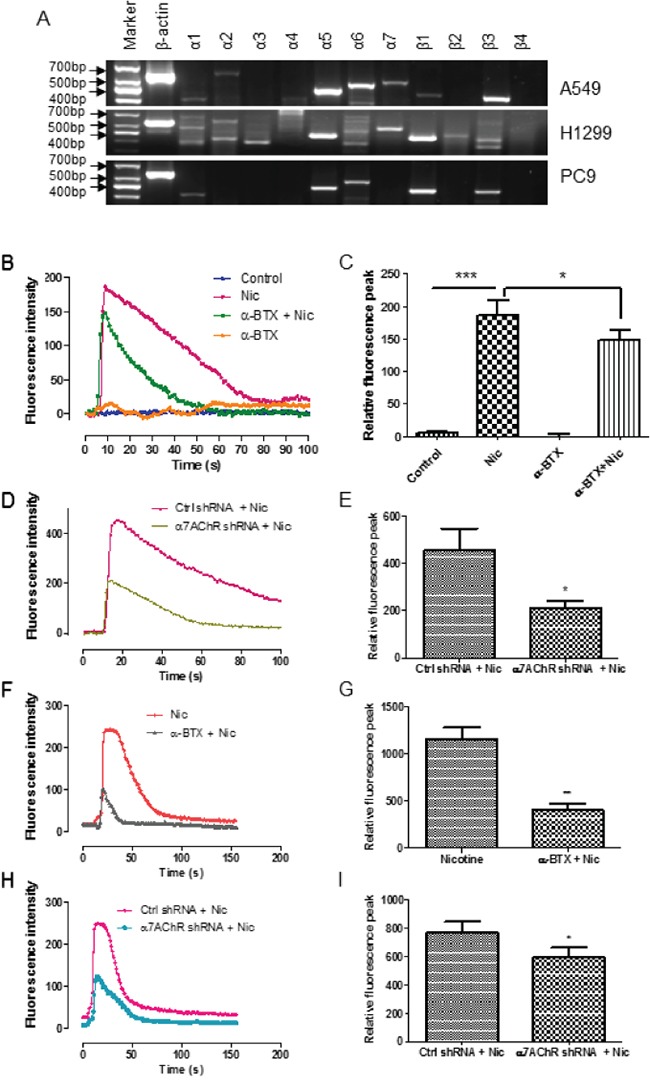
Functional expression of α7-nAChR in NSCLC cells **A.** RT-PCR analysis of nAChR subunits in A549, H1299, and PC9 cells. **B-I.** Measurement of calcium influx in A549 cells. The cells were treated with 1 mM nicotine and this effect was suppressed by the simultaneous administration of α-BTX (B and C) or by the knockdown of the α7 receptor subunit (D and E). Nicotine in 30 μM also can induce calcium influx and the effect was suppressed by the administration of α-BTX 60 min prior to the nicotine treatment (F and G) or by the knockdown of the α7 receptor subunit (H and I). The representative responses of the nicotine-induced increase of calcium influx measured by fluorescence intensity were shown (B, D, F, and H). Quantifications of the mean relative fluorescence peak calculated by [(F-F_0_)/F_0_]×100% are shown as means ± S.E.M from three independent experiments (C, E, G, I). ^*^ *P* < 0.05, ^**^ *P* < 0.01, ^***^ *P*< 0.001.

RT-PCR analysis showed the expression of the α7 subunit transcripts in A549 and H1299 cells but not in PC9 cells (Figure 1A). The α7 subunits formed functional nAChRs in the NSCLC cells, because the cells responded to nicotine by an increase of intracellular calcium influx (Figure 1B, 1C, 1D, 1E, 1F, 1G, and 1H) and the effect was hampered by the α7-nAChR selective antagonist α-bungarotoxin (α-BTX) (Figure 1B, 1C, 1F, and 1G) or by the knockdown of the α7 subunit via RNA interference (Figure 1D, 1E, 1H, and 1I).

**Figure 2:**
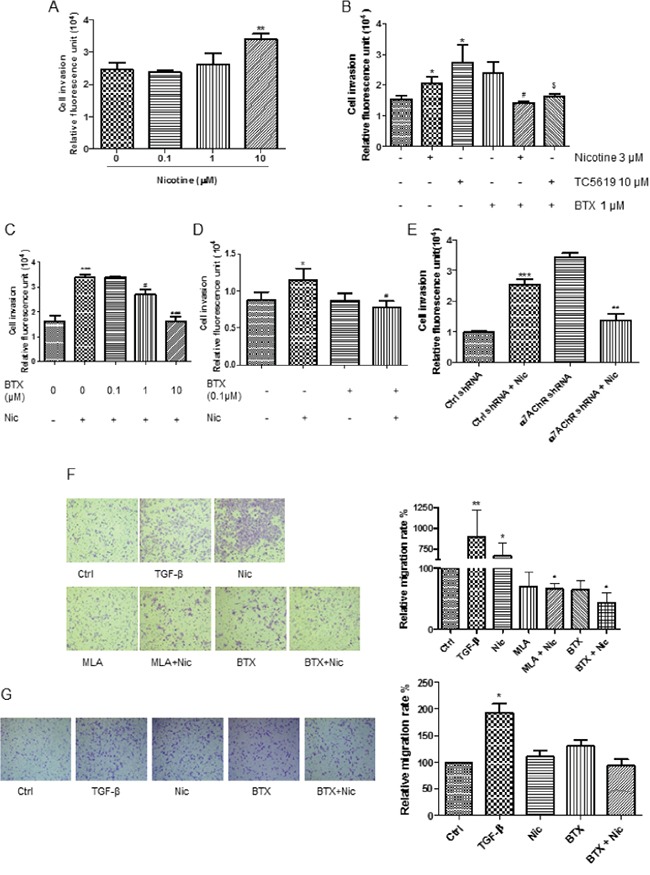
Dependence of α7-nAChR of nicotine-induced NSCLC cell invasion and migration **A.** Dose-dependence effect of nicotine on A549 cell invasion. The cells were treated for 48 h. **B.** Induction of A549 cell invasion by nicotine and α7-nAChR selectively agonist TC-5619 and the abrogation of the effect by α-BTX. *, *P* < 0.05 compared with the control group; #, *P* < 0.05 compared with the nicotine alone group; $, *P* < 0.05 compared with the TC5619 alone group. **C** and **D.** Blockade of nicotine-induced A549 cell invasion by α7-nAChR selectively antagonist α-BTX. When the antagonist was added simultaneously with the agonist, 1 μM of the antagonist was needed to attenuate the agonist-induced cell invasion (C); when the antagonist was added 1 h prior to the agonist, α-BTX at 0.1 μM fully blocked the effect (D). ^*^ *P* < 0.05, ^***^ *P*< 0.001 compared with the control group; #, *P* < 0.05, ###, *P* < 0.001 compared with the nicotine alone group. **E.** Attenuation of nicotine-induced A549 cell invasion by the knockdown of α7-receptor subunit. ***, *P* < 0.001 compared with the control group; ##, *P* < 0.01 compared with the nicotine-treated Control shRNA group. **F.** Abrogation of nicotine-induced A549 cell mobility by α7-nAChR selectively antagonist α-BTX and MLA. Images were taken with 10× objective lens. * P < 0.05, ** P < 0.01 compared with control group; # P < 0.05 compared with nicotine alone group. MLA, mecamylamine. TGF-β at 5 ng/mL as the migration-inducing positive control. The cells were treated by TGF-β or nicotine for 20 h; the antagonist α-BTX at 1 μM or MLA 1 μM was added 5 minutes prior to the agonists. **G.** Non-induction of PC9 cell mobility by nicotine. Images were taken with 10× objective lens. * P < 0.05 compared with control group. The cells were treated by TGF-β at 5 ng/mL or nicotine for 20 h; the antagonist α-BTX at 1 μM was added 5 minutes prior to the agonists. BTX, α-BTX. The cells were treated by 3 μM nicotine for 48 h in invasion assay and 1 μM nicotine for 20 h in migration assay unless otherwise indicated. Quantifications in bar graphs are shown as means ± S.E.M from at least three independent experiments.

Nicotine induced A549 cell invasion in a concentration-dependent manner (Figure 2A). Activation of α7-nAChR by TC5619, the subtype selective agonist, recapitulated the invasion-promotion effect (Figure 2B). The nicotine- or TC5619-induced cell invasion was abrogated by α-BTX (Figure 2B). α-BTX abrogated the nicotine-induced cell invasion in a concentration-dependent manner, with 10 μM having the maximum effect when the antagonist was concurrently administrated with the agonist (Figure 2C). When the antagonist was introduced 1 hour prior to the agonist, α-BTX at the concentration down to 0.1 μM can fully abolish the nicotine-induced cell invasion (Figure 2D). The α7-nAChR dependence of nicotine-induced invasion was reconfirmed in the α7-receptor subunit knockdown assay (Figure 2E). Analogously, nicotine induced A549 cell migration and the effect was abrogated by the α7-nAChR specific antagonists α-BTX and mecamylamine (MLA) (Figure 2F). PC9 cells, which lacked α7 subunit expression, failed to respond to nicotine in the induction of migration though they were sensitive to TGF-β (Figure 2G). These indicate that α7-nAChR mediates nicotine-induced NSCLC cell invasion and migration.

### α7-nAChR mediates nicotine-induced EMT in NSCLC cells

**Figure 3:**
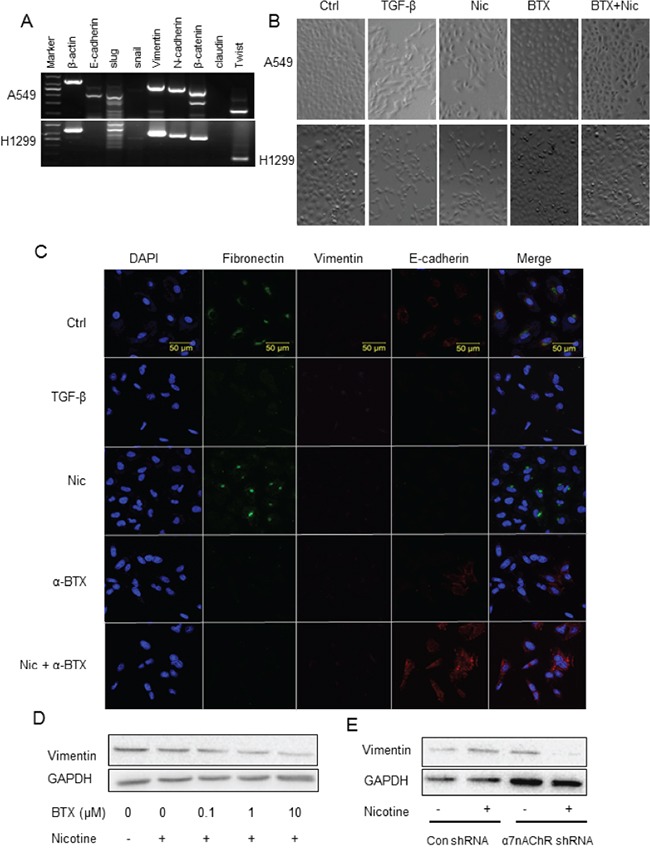
Dependence of α7-nAChR of nicotine-induced NSCLC cell EMT **A.** RT-PCR analysis of epithelial/mesenchymal markers in A549 and H1299 cells. **B.** Mesenchymal transition of A549 and H1299 cells stimulated by nicotine and the attenuation of the effect by α-BTX assayed by morphology analysis. TGF-β at 5 ng/mL as the EMT-inducing positive control. Images were taken with 10 × objective lens. **C.** Mesenchymal transition of A549 cells stimulated by nicotine and the attenuation of the effect by α-BTX assayed by immunofluorescence analysis of EMT protein markers. TGF-β at 5 ng/mL as the EMT-inducing positive control. Fibronectin and vimentin as the mesenchymal markers and E-cadherin as the epithelial marker. **D.** Down-regulation of vimentin expression in A549 cell by α7-nAChR antagonism assayed by western blot analysis. **E.** Attenuation of nicotine-induced upregulation of vimentin expression in A549 cells by knockdown of α7-nAChR subunit. The cells were treated by TGF-β or nicotine for 48 h; the antagonist α-BTX was added 5 minutes prior to the agonist.

Cell invasion and migration are closely involved in the process of EMT. We then investigated the effects of nicotine on the EMT of NSCLC cells and the receptor subtype mechanism. RT-PCR analysis showed the transcription of the epithelial marker E-cadherin in A549 cells and the mesenchymal markers vimentin, slug, N-cadherin, β-catenin, and twist in A549 cells and H1299 cells (Figure 3A).

Nicotine induced the EMT of the NSCLC cells. After nicotine treatment at 1 μM for 48 h, A549 and H1299 cells changed their morphology, including loss of apical-basal polarity, disappearance of cell-to-cell contacts, and front-to-back polarized compared with the untreated cells which showed more circular in shape and in greater cell-to-cell adhesion. The morphology change was blocked by the pre-treatment of α-BTX (Figure 3B). Immunofluorescent analysis showed that nicotine induced an up-regulation of mesenchymal marker fibronectin and down-regulation of epithelial marker E-cadherin expression in A549 cells (Figure 3C); the effect was blocked by the pre-treatment of α-BTX (Figure 3C). Western blot analysis showed that α-BTX decreased the expression of mesenchymal marker vimentin in a concentration-dependent manner (Figure 3D). The α7-nAChR dependence of nicotine-induced EMT was reconfirmed in the α7-subunit knockdown assay; nicotine induced up-regulation of the expression of the mesenchymal markers in Control shRNA cells whereas the α7-nAChR shRNA cells were resistant to this effect (Figure 3E).

### MEK/ERK signaling is involved in nicotine-induced invasion/migration

**Figure 4:**
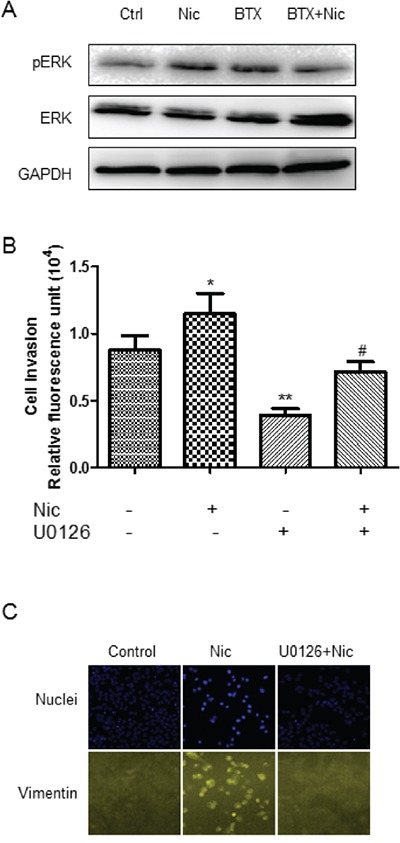
Involvement of MEK/ERK signaling in nicotine-induced invasion/migration **A.** Induction of ERK phosphorylation by nicotine and the attenuation of the effect by α7-nAChR antagonism. **B.** Attenuation of nicotine-induced A549 cell invasion by MEK-ERK selective inhibitor U0126. * P < 0.05, ** P < 0.01 compared with control group; # P < 0.05 compared with nicotine alone group. **C.** Attenuation of nicotine-induced vimentin upregulation by MEK-ERK selective inhibitor U0126. The cells were treated by 3 μM nicotine for 30 min in western blot analysis and for 48 h in invasion and HCA assay; U0126 at 50 μM was added 5 minutes prior to the agonist. Quantifications in bar graphs are shown as means ± S.E.M from at least three independent experiments.

The role of ERK in MEK/ERK signaling pathway in nicotine-induced invasion has not been well understood. Western blot showed that nicotine induced an up-regulation of ERK phosphorylation, indicating an activation of MEK/ERK signaling pathway (Figure 4A). The activation of MEK/ERK signaling was attenuated by α7-nAChR selective antagonism (Figure 4A). Inhibition of MEK/ERK pathway by U0126 decreased the nicotine-induced increase of cell invasion as shown in ECMatrix-based assay (Figure 4B) and abrogated nicotine-induced up-regulation of vimentin as shown in high-content analysis (HCA) (Figure 4C).

## DISCUSSION

Delineating the biological and pharmacological effect of nicotine on cancer cells at receptor subtype level is valuable for cancer prevention and treatment. In this study we have identified a facilitating effect of nicotine on NSCLC cell invasion, migration, and EMT and determined that the effect is mediated by the α7-nAChR involving MEK/ERK signaling pathway.

Metastasis is the major cause of cancer-related death. Invasion represents the metastatic step of the migration of cancer cells away from the primary site [9]. Better understanding of the nicotinic interference of NSCLC cell migration and invasion at receptor subtype level offers potentials for the exploitation of selective ligands for the control of tumor metastasis.

nAChR contains five subunits which assemble into heteromeric or homomeric pentamers. Different combinations of the subunits form different receptor subtypes and have distinct biophysical and pharmacological characteristics. While heteromeric α4β2-receptor and α5-receotpr exert negative effect on cell proliferation and migration, α7-nAChR has positive effect [8, 10]. This also might be one of the reason that nicotine seemed to inhibit the cell invasion and ERK phosphorylation when α7 receptor was blocked in the study; in this situation, the effect of other receptor subtypes such as α5 might be prominent. In keratinocytes, α3β2-nAChR and α7-nAChR are pivotal for nicotinergic chemokinesis, and α9-nAChR is critical for adhesion and motility [11]; in breast epithelial cells α9-nAChR is responsible for nicotine-stimulated cell growth and malignant transformation [12]. In our study, α7-nAChR specific antagonists and α7-nAChR knockdown completely abolished the nicotine-induced invasion, indicating the importance of α7-nAChR subtype in this process. This is consistent with the finding that nicotine promotes metastasis of pancreatic and colon cancers via α7nAChRs [13, 14].

The α7-nAChRs have the greatest Ca^2+^ permeability [15] and markedly affect cell invasion, migration, and EMT. Elevated cytosolic calcium activates PKC and subsequent MEK-ERK signaling cascade [10, 16–20]. On the other hand, α7-nAChR indirectly stimulates Raf-ERK signaling via facilitating the epidermal growth factor (EGF) release and the transactivation of EGF receptors [21]. ERK signaling pathway dictates EMT and invasion. Cooperating with TGF-β signaling pathways, ERK signaling cascade upregulates the expression of the mesenchymal genes and epithelial repressor genes [22]. Our study identified that nicotine via α7-nAChRs activated ERK signaling and upregulated the expression of mesenchymal protein fibronectin and vimentin in NSCLC cells, indicating the role of the receptor subtype in the EMT process involving MEK/EKR signaling pathway.

EMT represents a process that cells lose epithelial properties and obtain more migratory mesenchymal characteristics. This process depends on the up-regulation of mesenchymal proteins vimentin and fibronectin. Nicotine induced the EMT and up-regulation of vimentin in NSCLC cells and the effects were suppressed by α7-nAChR selective antagonist α-BTX and α7-nAChR knockdown. Similar effect of nicotine on EMT has also been identified in breast, colon, and gastric cancer cells [6, 14, 23]. In carcinoma cells, the acquired mesenchymal properties from the EMT process donate these cells the various traits required to implement multiple steps of the invasion-metastasis cascade by facilitating the cells with the ability to migrate, invade away from the primary site, travel through the blood and lymph vessels, and eventually make up metastases [24].

The concentration of nicotine that markedly induces NSCLC cell invasion and EMT ranges from 0.1-10 μM in our and other studies. The nicotine concentrations in smoker blood fall in this range. The average steady state level is about 0.2 μM and spikes to 10 μM or more right after cigarette smoking [25, 26]. In addition to causing cancers, nicotine leads to tobacco addiction, making the smokers consume more cigarettes and exposed to more nicotine. These add the warrant of quitting active or passive smoking and the rational of nAChRs as the target for cancer control.

Taking together, nicotine induces NSCLC cell invasion, migration, and EMT; the effects are mediated by α7-nAChRs and involve MEK/ERK signaling pathway. This finding would improve the understanding of cancer biology and offer potentials for the exploitation of selective ligands for the control of the cancer metastasis.

## MATERIALS AND METHODS

### Reagents

Nicotine, α-BTX, MLA and TC5619 were purchased from Sigma-Aldrich (St. Louis, MO, USA). U0126 was purchased from Beyotime Biotechnology (Shanghai, China). Antibodies used for Western blot or immunofluorescence were purchased from Cell Signaling Technology (Danvers, MA, USA) for ERK, phosphor-p42/44 ERK, vimentin, E-cadherin, fibronectin and GAPDH.

### Cell culture

Human NSCLC cell lines A549 and H1299 were purchased from the Chinese Academy of Sciences Cell Bank of Type Culture Collection (Shanghai, China). PC9 cell line was kindly gifted by Professor Qiang-Gang Dong in Shanghai Cancer Institute affiliated to Shanghai Jiao Tong University. A549, H1299, and PC9 cells were cultured in 10% fetal bovine serum (FBS)-contained F12K, RPMI1640, and high glucose DMEM medium (Invitrogen, Carlsbad, CA, USA), respectively, in 37°C with 5% CO_2_.

### Migration assay

Cell migration assays were performed in 24-well transwell Boyden chambers (8 μm pore size; Costar, MA, USA) according to the vendor's instructions. Briefly, 100 μL cell suspension (without serum) containing 5×10^5^ cells/mL was seeded into the upper migration chamber. A 600 μl medium containing 10% FBS which served as the chemo-attractant was added to the lower chambers of the transwell plates. After incubation in 37°C for 20 h, the media in the lower chamber were discarded. A 600 μL 1% glutaraldehyde was added to the lower chambers to fix the cells on the membrane for 15 min at room temperature. The upper chambers were removed and wiped with a cotton swab to clear the residual cells on the upper side. Cells migrating to the lower surface of the polycarbonate membrane were stained with 0.1% crystal violet for 25 min. The membranes were rinsed with Dulbecco's phosphate-buffered saline (DPBS) buffer and photographed by using a microscope (Olympus, DP50, Tokyo, Japan). The areas of cell migration were quantified by counting 3 randomly selected microscopic fields and the digital pixel densitometry of images was analyzed using Image-J software (National Institutes of Health, USA).

### Invasion assay

The QCM ECMatrix™ based 24-well cell invasion assay kit was used to assess tumor cell invasion according to the manufacturer's protocol (8 μm pore size; Millipore Inc, USA). The kit utilizes ECMatrix, a reconstituted basement membrane matrix of proteins derived from the Engelbreth Holm-Swarm (EHS) mouse tumor. The ECM layer occludes the membrane pores, blocking the migration of non-invasive cells. Invasive cells, on the other hand, migrate through the ECM layer and cling to the bottom of the polycarbonate membrane. Cell suspensions (0.5 ~ 4.0 × 10^5^/mL) were loaded to the interior of the inserts in FBS-free F12K- or RPMI1640-medium. The lower chambers were filled with medium containing 10% FBS serving as the chemo-attractant. The plates were covered and incubated for 48 hours at 37°C in a 5%CO_2_ incubator. The remaining cell suspensions containing non-invaded cells were carefully removed from the upper side of the insert with a pipette. The invasion chamber inserts were placed into clean wells containing 225 μL of pre-warmed cell detachment solution and incubated for 30 minutes at 37°C. The cells invading the ECMatrix™-coated membrane on the bottom of the insert membrane were dissociated from the membrane when incubated with the cell detachment buffer supplied with the kit. Those cells were subsequently lysed and detected by the patented CyQuant GR dye which exhibited strong fluorescence enhancement when bound to cellular nucleic acids. The mixture was read with a fluorescence plate reader using 480/520 nm filter set to measure quantitatively the fluorescence intensity of the invading cells.

### RNA extraction and reverse transcriptase-polymerase chain reaction (RT-PCR)

Total RNAs were extracted from cells using the E.Z.N.A. DNA/RNA/Protein isolation kit (Omega Bio-Tek, USA) according to the manufacturer's instructions. Reverse transcriptions were carried out using a Quantscript RT kit (TIANGEN, China), and the resultant single strand cDNAs were stored at −20°C for later use in the PCR reactions. PCRs were performed with TaKaRa Ex Taq (TaKaRa Biotechnology, China). The cDNAs were amplified according to the following temperature profile: 94°C for 30 s, 55°C for 30 s, and 72°C for 1 min. At the end of 31 cycles, the reaction was continued for an additional 10 min at 72°C, and PCR products (24 μL) were analyzed electrophoretically on 2% agarose gels using 1 × TAE buffer. The primer sequences used for PCR are shown in Supplementary Tables S1.

### Protein extraction and Western-blot analysis

Protein levels were measured as following. Briefly, all the cultured cells were rinsed with ice-cold PBS, lysed in 150 or 200 μL lysis buffer RIPA containing 1 mM PMSF (Beyotime, China) on ice. Lysates were collected after centrifugation at 14000 r.p.m for 8 min at 4°C. The supernatants were collected and the protein concentrations were determined using BCA Protein Assay Kit (Pierce Chemical, Rockford, IL, USA). The lysates were solubilized with 5×SDS-PAGE sample loading buffer (Beyotime, China) and boiled for 5 min at a temperature of 100°C to denature the protein. Equal amounts of protein extracts were separated on 10% sodium dodecyl sulfate (SDS)-polyacrylamide gels electrophoresis (PAGE). Subsequently, the separated proteins were transferred to polyvinylidinedifluoride (PVDF) membranes (Millipore Billerica, MA, USA) by electroblotting. Membranes were blocked with 5% nonfat dry milk in Tris-buffered saline and 0.1% Tween 20 (TBST) for 1 hour at room temperature, washed for 5 minutes with wash buffer (1×TBST) and incubated overnight at 4°C with the following primary antibodies: rabbit anti-vimentin, ERK1/2, and GAPDH (1:1000), rabbit anti-p-ERK1/2 (1:2000). Membranes were then washed with TBST several times, and incubated with secondary antibodies including goat anti-rabbit IgG conjugated to horseradish peroxidase (1:1000) for 1 hour at room temperature. The immunoblots were then visualized and scanned using the Odyssey FC Imaging System (LI-COR Biosciences, NE, USA).

### Transfection of shRNA

Human α7-nAChR shRNA lentiviral particles (sc-42532-V) and control shRNA lentiviral particles (sc-108080) were purchased from Santa Cruz, USA. The shRNA transfection was performed according to the manufacturer's instructions. Each milliliter medium contained 5 × 10^4^ infectious units of virus. Cells with stable integration of shRNA were chosen to use with 10 μg/mL puromycin

### Immunofluorescence, confocal microscopy and high-content analysis

Cells were detached, seeded onto multiple glass-bottom tissue culture plates (10 mm, Shengyou Biotechnology, China) and cultured for 24 h with complete medium containing 10% FBS. Sub-confluent cells were washed with PBS rapidly at room temperature (RT). Cells were fixed in 4% paraformaldehyde for 15 min at 37°C, washed in cold PBS and blocked for 30 min with 1% FBS in PBS at RT. Cells were then washed and incubated with primary antibody at the concentration of 1: 100 overnight at 4°C in the wet dark box. The primary antibodies used were as following: anti-vimentin, 1:100; anti-E-cadherin, 1:100 and anti-fibronectin, 1:100. Next, cells were washed and stained with FITC-conjugated secondary antibodies (1:100) at 37°C for 1 h, and then washed with PBS. The samples were mounted in 1:2000 DAPI and analyzed using confocal laser scanning microscope Zeiss LSM 710 (Zeiss, Thornwood, NY, USA) or HCA (ArrayScan XTI, Thermo Scientific).

### Calcium influx

The cells were seeded onto the glass chamber slides (Shengyou Biotechnology, 043320B; China). After culturing for 24 hours, the medium was removed and the cells were incubated with 1 μM solution of Fluo-4 AM (DojindDo, China) in Hank's balanced salt solution (HBSS) at 37°C for 1h. Cells were then washed once with HBSS and stored in HBSS for 30 minutes before analysis and imaged with a Zeiss LSM-510 (Germany) EXCITER microscope.

### Statistical analysis

Student's t-test or one-way ANOVA with Bonferroni post-test was properly used to test statistical significance with GraphPad Prism5.0 software (La Jolla, CA, USA). Differences were considered significant if P value was less than 0.05.

## SUPPLEMENTARY TABLE



## References

[1] DeSantis CE, Lin CC, Mariotto AB, Siegel RL, Stein KD, Kramer JL, Alteri R, Robbins AS, Jemal A (2014). Cancer treatment and survivorship statistics 2014. CA Cancer J Clin.

[2] Edwards BK, Noone AM, Mariotto AB, Simard EP, Boscoe FP, Henley SJ, Jemal A, Cho H, Anderson RN, Kohler BA, Eheman CR, Ward EM (2014). Annual Report to the Nation on the status of cancer 1975-2010 featuring prevalence of comorbidity and impact on survival among persons with lung colorectal breast or prostate cancer. Cancer.

[3] Inamura K, Ishikawa Y (2010). Lung cancer progression and metastasis from the prognostic point of view. Clin Exp Metastasis.

[4] Dresler C (2013). The changing epidemic of lung cancer and occupational and environmental risk factors. Thorac Surg Clin.

[5] Cardinale A, Nastrucci C, Cesario A, Russo P (2012). Nicotine: specific role in angiogenesis proliferation and apoptosis. Crit Rev Toxicol.

[6] Dasgupta P, Rizwani W, Pillai S, Kinkade R, Kovacs M, Rastogi S, Banerjee S, Carless M, Kim E, Coppola D, Haura E, Chellappan S (2009). Nicotine induces cell proliferation invasion and epithelial-mesenchymal transition in a variety of human cancer cell lines. Int J Cancer.

[7] Puisieux A, Brabletz T, Caramel J (2014). Oncogenic roles of EMT-inducing transcription factors. Nat Cell Biol.

[8] Krais AM, Hautefeuille AH, Cros MP, Krutovskikh V, Tournier JM, Birembaut P, Thepot A, Paliwal A, Herceg Z, Boffetta P, Brennan P, Hainaut PL (2011). CHRNA5 as negative regulator of nicotine signaling in normal and cancer bronchial cells: effects on motility migration and p63 expression. Carcinogenesis.

[9] Clark AG, Vignjevic DM (2015). Modes of cancer cell invasion and the role of the microenvironment. Curr Opin Cell Biol.

[10] Schuller HM (2007). Neurotransmitter receptor-mediated signaling pathways as modulators of carcinogenesis. Prog Exp Tumor Res.

[11] Chernyavsky AI, Arredondo J, Marubio LM, Grando SA (2004). Differential regulation of keratinocyte chemokinesis and chemotaxis through distinct nicotinic receptor subtypes. J Cell Sci.

[12] Lee CH, Huang CS, Chen CS, Tu SH, Wang YJ, Chang YJ, Tam KW, Wei PL, Cheng TC, Chu JS, Chen LC, Wu CH, Ho YS (2010). Overexpression and activation of the alpha9-nicotinic receptor during tumorigenesis in human breast epithelial cells. J Natl Cancer Inst.

[13] Momi N, Ponnusamy MP, Kaur S, Rachagani S, Kunigal SS, Chellappan S, Ouellette MM, Batra SK (2013). Nicotine/cigarette smoke promotes metastasis of pancreatic cancer through alpha7nAChR-mediated MUC4 upregulation. Oncogene.

[14] Wei PL, Kuo LJ, Huang MT, Ting WC, Ho YS, Wang W, An J, Chang YJ (2011). Nicotine enhances colon cancer cell migration by induction of fibronectin. Ann Surg Oncol.

[15] Fucile S (2004). Ca2+ permeability of nicotinic acetylcholine receptors. Cell Calcium.

[16] Carlisle DL, Liu X, Hopkins TM, Swick MC, Dhir R, Siegfried JM (2007). Nicotine activates cell-signaling pathways through muscle-type and neuronal nicotinic acetylcholine receptors in non-small cell lung cancer cells. Pulm Pharmacol Ther.

[17] Heeschen C, Jang JJ, Weis M, Pathak A, Kaji S, Hu RS, Tsao PS, Johnson FL, Cooke JP (2001). Nicotine stimulates angiogenesis and promotes tumor growth and atherosclerosis. Nat Med.

[18] Heusch WL, Maneckjee R (1998). Signalling pathways involved in nicotine regulation of apoptosis of human lung cancer cells. Carcinogenesis.

[19] Improgo MR, Tapper AR, Gardner PD (2011). Nicotinic acetylcholine receptor-mediated mechanisms in lung cancer. Biochem Pharmacol.

[20] Trombino S, Bisio A, Catassi A, Cesario A, Falugi C, Russo P (2004). Role of the non-neuronal human cholinergic system in lung cancer and mesothelioma: possibility of new therapeutic strategies. Curr Med Chem Anticancer Agents.

[21] Chernyavsky AI, Shchepotin IB, Grando SA (2015). Mechanisms of growth-promoting and tumor-protecting effects of epithelial nicotinic acetylcholine receptors. Int Immunopharmacol.

[22] Neuzillet C, Tijeras-Raballand A, de Mestier L, Cros J, Faivre S, Raymond E (2014). MEK in cancer and cancer therapy. Pharmacol Ther.

[23] Wang W, Chin-Sheng H, Kuo LJ, Wei PL, Lien YC, Lin FY, Liu HH, Ho YS, Wu CH, Chang YJ (2012). NNK enhances cell migration through alpha7-nicotinic acetylcholine receptor accompanied by increased of fibronectin expression in gastric cancer. Ann Surg Oncol.

[24] Polyak K, Weinberg RA (2009). Transitions between epithelial and mesenchymal states: acquisition of malignant and stem cell traits. Nat Rev Cancer.

[25] Armitage AK, Dollery CT, George CF, Houseman TH, Lewis PJ, Turner DM (1975). Absorption and metabolism of nicotine from cigarettes. Br Med J.

[26] Russell MA, Jarvis M, Iyer R, Feyerabend C (1980). Relation of nicotine yield of cigarettes to blood nicotine concentrations in smokers. Br Med J.

