# Supplementary material for: Role of α7-nicotinic acetylcholine receptor in nicotine-induced invasion and epithelial-to-mesenchymal transition in human non-small cell lung cancer cells
Source: Oncotarget. 2016 Jul 8;7(37):59199–208. doi: 10.18632/oncotarget.10498 (PMC5312305; doi:10.18632/oncotarget.10498)
Supplement: Supplementary file 1 [file oncotarget-07-59199-s001.pdf]

Role of α7-nicotinic acetylcholine receptor in nicotine-induced invasion and epithelial-to-mesenchymal transition in human non-small cell lung cancer cells

SUPPLEMENTARY TABLE

Supplementary Table S1: The primer sequences used for PCR

A. The primer sequences used for PCR of nAChR subunits

Gene	Primer Sequence (5'- 3')	Fragment (bp)
α1NAChR_F	gaatgagcaatgggtggattac	452
α1NAChR_R	tgacgaagtggtaggtgatgtc	
α2NAChR_F	gtctggctaaaacaggagtggag	499
α2NAChR_R	ggatgatgaggttgatggtgta	
α3NAChR_F	tggtgaaggtggatgaagtaaa	490
α3NAChR_R	gagtatgtgatgtcggggtaga	
α4NAChR_F	ggatgagaagaaccagatgatga	487
α4NAChR_R	gacgaaggcataggtgatgtc	
α5NAChR_F	atggagaatgggagattgtgag	386
α5NAChR_R	tgaggaagaacgatgatgaatg	
α6NAChR_F	caagtagaaggcaaaacaaaagc	464
α6NAChR_R	aacacagtcagagaaagcaggac	
α7-nAChR_F	tgactcgcaaccactcacc	499
α7-nAChR_R	gcactcatagaacctttcactcc	
β1NAChR_F	cctgctgacccttactgtgttc	363
β1NAChR_R	ggtttggggaagagaaaatcac	
β2NAChR_F	ctggaagcctgaagagtttgac	452
β2NAChR_R	ggatgatgaggttgatggtgta	
β3NAChR_F	cggagaatgggaaatactgaac	354
β3NAChR_R	aacacggtaacaatgatggaca	
β4NAChR_F	aacaggaatggactgattaccg	480
β4NAChR_R	ggatgatgaggttgatggtgta	
β-ACTIN_F	ctgggacgacatggagaaaa	434
β-ACTIN_R	aaggaaggctggaagagtgc	

B. Primer sequences used for genes of epithelial and mesenchymal markers

Gene	Primer Sequence (5'- 3')	Fragment(bp)
Cdh1_F1	cttctgctgatcctgtctgatg	322
Cdh1_R1	agtcacacacgctgacctctaa	
VIM_F	tacaaatccaagtttgctgacct	500
VIM_R	tcgttgataacctgtccatctct	
SNAI2_F1	caaaaccagagatcctcacctc	349
SNAI2_R1	gatttcctagactgggcatcac	
SNAI1_F1	atccagagtttaccttccagca	409
SNAI1_R1	tccttgttgcagtatttgcagt	
CTNNB1_F1	tcccaagtcctgtatgagtgg	414
CTNNB1_R1	agcagacaccatctgaggaga	
CDH2_F	gggtaatcctcccaaatcaaat	455
CDH2_R	taatgaagtccccaatgtctcc	
CLDN1_F1	agaagatgaggatggctgtca	249
CLDN1_R1	ttggtgttgggtaagaggttg	
TWIST1_F1	gtccgcagtcttacgaggag	148
TWIST1_R1	gtctgaatcttgctcagcttgtc	
CLDN1_F1	agaagatgaggatggctgtca	249
CLDN1_R1	ttggtgttgggtaagaggttg	
TWIST1_F1	gtccgcagtcttacgaggag	148
TWIST1_R1	gtctgaatcttgctcagcttgtc	
β-actin_F	ctgggacgacatggagaaaa	434
β-actin_R	aaggaaggctggaagagtgc	
